# A Data Integration Multi-Omics Approach to Study Calorie Restriction-Induced Changes in Insulin Sensitivity

**DOI:** 10.3389/fphys.2018.01958

**Published:** 2019-02-05

**Authors:** Maria Carlota Dao, Nataliya Sokolovska, Rémi Brazeilles, Séverine Affeldt, Véronique Pelloux, Edi Prifti, Julien Chilloux, Eric O. Verger, Brandon D. Kayser, Judith Aron-Wisnewsky, Farid Ichou, Estelle Pujos-Guillot, Lesley Hoyles, Catherine Juste, Joël Doré, Marc-Emmanuel Dumas, Salwa W. Rizkalla, Bridget A. Holmes, Jean-Daniel Zucker, Karine Clément, The MICRO-Obes Consortium, Aurélie Cotillard

**Affiliations:** ^1^Sorbonne University, French National Institute for Health and Medical Research, NutriOmics Unit, Institute of Cardiometabolism and Nutrition, Paris, France; ^2^Danone Nutricia Research, Palaiseau, France; ^3^Institute of Cardiometabolism and Nutrition, Integromics, ICAN, Paris, France; ^4^Sorbonne University, IRD, UMMISCO, Bondy, France; ^5^Section of Biomolecular Medicine, Division of Integrative Systems Medicine and Digestive Disease, Department of Surgery and Cancer, Faculty of Medicine, Imperial College London, London, United Kingdom; ^6^Assistance Publique Hôpitaux de Paris, Nutrition Department, CRNH Ile-de-France, Pitié-Salpêtrière Hospital, Paris, France; ^7^Institute of Cardiometabolism and Nutrition, ICANalytics, Paris, France; ^8^Institut National de la Recherche Agronomique, Unité de Nutrition Humaine, Plateforme d’Exploration du Métabolisme, MetaboHUB, Université Clermont Auvergne, Clermont-Ferrand, France; ^9^Department of Bioscience, School of Science and Technology, Nottingham Trent University, Clifton Campus, Nottingham, United Kingdom; ^10^National Institute of Agricultural Research, Micalis Institute, AgroParisTech, Université Paris-Saclay, Jouy-en-Josas, France

**Keywords:** data integration, insulin sensitivity, lifestyle factors, microbiota, omics

## Abstract

**Background:** The mechanisms responsible for calorie restriction (CR)-induced improvement in insulin sensitivity (IS) have not been fully elucidated. Greater insight can be achieved through deep biological phenotyping of subjects undergoing CR, and integration of big data.

**Materials and Methods:** An integrative approach was applied to investigate associations between change in IS and factors from host, microbiota, and lifestyle after a 6-week CR period in 27 overweight or obese adults (ClinicalTrials.gov: NCT01314690). Partial least squares regression was used to determine associations of change (week 6 – baseline) between IS markers and lifestyle factors (diet and physical activity), subcutaneous adipose tissue (sAT) gene expression, metabolomics of serum, urine and feces, and gut microbiota composition. ScaleNet, a network learning approach based on spectral consensus strategy (SCS, developed by us) was used for reconstruction of biological networks.

**Results:** A spectrum of variables from lifestyle factors (10 nutrients), gut microbiota (10 metagenomics species), and host multi-omics (metabolic features: 84 from serum, 73 from urine, and 131 from feces; and 257 sAT gene probes) most associated with IS were identified. Biological network reconstruction using SCS, highlighted links between changes in IS, serum branched chain amino acids, sAT genes involved in endoplasmic reticulum stress and ubiquitination, and gut metagenomic species (MGS). Linear regression analysis to model how changes of select variables over the CR period contribute to changes in IS, showed greatest contributions from gut MGS and fiber intake.

**Conclusion:** This work has enhanced previous knowledge on links between host glucose homeostasis, lifestyle factors and the gut microbiota, and has identified potential biomarkers that may be used in future studies to predict and improve individual response to weight-loss interventions. Furthermore, this is the first study showing integration of the wide range of data presented herein, identifying 115 variables of interest with respect to IS from the initial input, consisting of 9,986 variables.

**Clinical Trial Registration:** clinicaltrials.gov (NCT01314690).

## Introduction

Obesity-associated impairment of glucose homeostasis is influenced by multiple elements including diet, physical activity, pharmacology and other lifestyle factors, predisposition from the host due to genetics, epigenetics, physiology and, as discovered more recently, gut microbiota alterations (Mutch and Clément, 2006; Khan et al., 2014; Mozaffarian, 2016). Although these elements have been implicated in different facets of the etiology of type 2 diabetes (T2D), specific mechanisms showing how they interact remain partially understood. Through high-throughput generation of biological data and analytical approaches involving data integration of elements from multiple sources, we endeavored to shed light on these complex interactions and highlight relevant mechanistic pathways.

Gut microbiota composition and function may play an important role in the prevention but also progression of insulin resistance (Qin et al., 2012; Cotillard et al., 2013; Karlsson et al., 2013; Le Chatelier et al., 2013; Dao et al., 2015; Forslund et al., 2015; Pedersen et al., 2016). Some bacterial species, such as *Akkermansia muciniphila* and *Faecalibacterium prausnitzii*, as well as high gut microbial gene richness are known to be associated with better metabolic and intestinal health (Cotillard et al., 2013; Le Chatelier et al., 2013; Dao et al., 2015; Plovier et al., 2017). Interestingly, studies considering the microbiome’s functional potential have identified a series of pathways that are linked with different stages of insulin resistance. Among these pathways, branched chain amino acid (BCAA) metabolism by microbiota stands out. A recent study of the metagenome found increased abundance of bacterial genes coding for BCAA production and decreased abundance of genes coding for BCAA transporters on the bacterial wall in relation to insulin resistance in non-diabetic Danish adults (Pedersen et al., 2016).

High circulating concentrations of BCAA may disrupt glucose homeostasis in situations of stress on the body such as in high caloric intake (especially high-fat diets) or diets rich in foods with high glycemic index and excess body weight (Newgard et al., 2009; Roberts et al., 2014). BCAA have been repeatedly found to be elevated in T2D and obesity, and have been considered as early predictors of T2D (Roberts et al., 2014). Through activation of the nutrient-sensitive mTOR pathway and shift toward anabolism, BCAA may be redirected from protein synthesis toward gluconeogenesis with disruption of glucose homeostasis partly through chronically increased insulin secretion. Excessive fat mass storage in obesity may lead to not only *in situ* metabolic disruption, with some evidence of decreased BCAA catabolism (Roberts et al., 2014), but also ectopic fat deposition and inflammation. These modifications play an important role in the development of insulin resistance.

Lifestyle interventions resulting in weight loss, which involve calorie restriction (CR) together with improved dietary quality, and increased physical activity, lead to amelioration of IS (Després et al., 2001; Speakman and Mitchell, 2011; Kong et al., 2013). However, response to these interventions greatly varies from one person to another and a greater understanding at the individual level is needed to predict and enhance response (Kong et al., 2013; Dao et al., 2016). CR has been shown to significantly modify the transcriptional gene profile in subcutaneous adipose tissue (sAT) (Clément et al., 2004; Viguerie et al., 2005; Capel et al., 2009; Rizkalla et al., 2012), as well as other AT depots, and to modulate microbiota composition and function (reviewed in Dao et al., 2016).

So far, knowledge about the relationship between lifestyle factors, host biology, gut microbiota and gut-derived metabolites is somewhat fragmented. A better understanding of their interconnection may be achieved through data integration approaches across different disciplines of biological sciences and medicine (Kussmann et al., 2013; Kidd et al., 2014; Wu et al., 2015; Dao et al., 2016), and great efforts to develop data integration methods are currently being undertaken. Data integration can provide greater biological insight, a better understanding of relationships between variables, and may lead to innovative data-driven hypotheses paving the way to the discovery of mechanistic links. In fact, new data integration approaches are increasingly required to analyze the vast amounts of data being collected from deep phenotyping of patients (Piening et al., 2018).

The goal of this study was to improve our understanding of the inter-connectivity of IS and lifestyle, gut microbiome and host factors through the use a data integration approach. We aimed to conduct an in-depth exploration of associations between CR-induced improvement in IS and changes in host biology, gut microbiota and lifestyle factors. Novel connections between these elements were identified.

## Materials and Methods

### Study Population and Dietary Intervention

A subgroup (*N* = 27) of the MICRO-Obes study (Cotillard et al., 2013; Kong et al., 2013, 2014) has been included in this report, based on AT sample availability. All analyses have been conducted using the delta (week 6 – baseline) of the different variables. The participants were overweight and obese adults [3 men and 24 women, median [interquartile range (IQR)] age and BMI: 41 (26) years and 33.9 (5.8) kg/m^2^] with no chronic conditions and no medication use. The dietary intervention was completed at the Pitié-Salpêtrière Hospital in Paris, France and consisted of a 6-week individually prescribed hypocaloric diet (1200 kcal/d for women and 1500 kcal/d for men) followed by a 6-week weight-maintenance period. Only information collected at baseline and week 6 of the intervention has been included in the present analysis. This diet consisted of habitually consumed foods plus meal replacement with four dietary products daily (60–75 kcal; designed by CEPRODI-KOT Laboratory). These supplements consisted of lyophilized powder enriched in protein and soluble fiber (mainly inulin) and composed of low glycemic index carbohydrates. This intervention has been previously described in detail (Rizkalla et al., 2012; Kong et al., 2013). This study has been registered in ClinicalTrials.gov (NCT01314690) and approved by the Ethical Committee of Hôtel-Dieu Hospital in Paris, France in 2008 (under the number 0811792). All participants provided written informed consent. Data collection occurred in 2009 and 2010.

### Assessment of Clinical Parameters

Body composition, sAT adipocyte diameter, and fasting blood lipids, insulin, glucose, inflammatory markers, and IS/secretion markers were assessed as described previously (Dao et al., 2015). Body composition measures included anthropometric measurements (weight, height, waist, and hip circumference), and dual energy X-ray (DXA) absorptiometry. Blood samples were collected after a 12-h fast.

### Assessment of Food and Drink Intake

Diet was assessed with a 7-day unweighted food diary administered at baseline and week 6, as previously described in Kong et al. (2014). The software PROFILE DOSSIER V3 was used for analysis of the diaries and consumed foods were grouped into 26 food groups according to French dietary surveys. Average intake of energy, nutrients, and food groups were calculated from intake over the 7 days. The PROFILE DOSSIER X029 computer software (Audit Conseil en Informatique Médicale, Bourges, France) was used to analyze all food diaries and generate nutrient intakes. This software has a food composition database consisting of 400 food items which are representative of the French diet (Bouché et al., 2002).

### Change in Diet and Clinical Variables

Changes in clinical and diet data were estimated using SAS 9.4. Median and IQR are reported for all variables. Statistical significance in change in clinical outcomes was determined with Wilcoxon signed rank sum test. Correction for multiple testing per class of variable was done using BH with FDR at the 5% level, and adjusted *P*-values are reported. *Post hoc* statistical power calculations for the change in IS markers from baseline to week 6 of CR is presented in Supplementary Table 1.

### Adipose Tissue Biopsies and Gene Expression With Microarray Analysis

Subcutaneous adipose samples were collected at baseline and week 6 by needle biopsy from the periumbilical region under local anesthesia. Samples were frozen rapidly in liquid nitrogen then stored at -80°C until they were further processed for microarray analysis as described in Rizkalla et al. (2012). Briefly, total RNA was extracted by using the RNeasy total RNA Mini kit (QIAGEN) with one-column DNase digestion. RNA quality and concentration were assessed by using an Agilent 2100 Bioanalyzer (Agilent Technologies). An Illumina RNA amplification kit (Ambion) was used according to the manufacturer’s instructions to obtain biotin-labeled complementary RNA from 250 ng total RNA. Hybridization processes were performed with Illumina Human HT-12 version 3.0 Expression BeadChips (Illumina, Inc.). Hybridized probes were detected with cyanin-3-streptavidin (1 mg/mL; Amersham Biosciences, GE Health Care) and scanned by using an Illumina BeadArray Reader. Raw data were extracted with GenomeStudio 2011.1 Software by using the default settings and quantile normalization. Relevant genes associated with changes in IS were annotated using the FunNet tool (Prifti et al., 2008), using the whole human genome as the reference gene set.

### Batch Correction of Microarray Data

Transcriptomics data were adjusted for batch effects that are technical artifacts inherent to DNA/RNA sample processing (Supplementary Figure 1A) (Leek et al., 2010). We chose the pSVA approach (Chen et al., 2011; Parker et al., 2014), to remove these effects based on an in-house micro-array quality control procedure. The pSVA adjustment method, which is based on singular value decompositions, has been shown to be very efficient in preserving biological heterogeneity (Parker et al., 2014).

We have evaluated the adjustment quality of our data following the approach described in Gagnon-Bartsch and Speed (Gagnon-Bartsch and Speed, 2012). Specifically, two sets of control genes have been determined from the literature and narrowed down by statistical analysis: positive controls (18 genes) that were shown to be differentially expressed between male and female AT, and negative controls (47 genes) whose expression is independent of the gender biological signal (Supplementary Figures 1B,C). *P*-value rank of controls were assessed before and after batch effect correction to identify the optimal adjustment approach (Supplementary Figures 1D,E). A complementary qPCR analysis confirmed the batch effect correction improvement (Supplementary Figures 1F,G). The final data set included only genes that were 100% present in all 27 subjects at both time points.

### Analysis of sAT Gene Expression by qPCR

RNAs from AT were prepared using RNeasy Lipid Tissue kit (Qiagen) and cDNAs were synthesized using SuperScript II and random hexamers (Promega). Quantitative PCR with SybrGreen was performed and relative quantification of each transcript in comparison to 18S was determined using the 2^-ΔΔCt^ method. All primer sequences are available upon request.

### Metagenomic Sequencing for Assessment of Gut Microbiota

Fecal microbiota was characterized with shotgun quantitative metagenomics (*N* = 27). Total fecal DNA was analyzed with high throughput SOLiD sequencing, as described in Cotillard et al. (2013). Reads were mapped and counted onto the 3.9 million gene catalog, as described in Dao et al. (2015). Only MGS with more than 700 genes were considered.

### Serum and Urine Metabolic Phenotyping by ^1^H NMR Spectroscopy

Urine (*N* = 21) and serum (*N* = 24) samples were randomized, prepared and measured on a NMR spectrometer (Bruker) operating at 600.22 MHz ^1^H frequency using previously published experimental parameters (Dona et al., 2014). The ^1^H NMR spectra were then pre-processed and analyzed as previously reported (Dumas et al., 2006) using Statistical Recoupling of Variables-algorithm (Blaise et al., 2009). Structural assignment was performed as reviewed in Dona et al. (2016), using in-house and publicly available databases.

### Fecal Metabolomics

Analysis of frozen fecal samples (*N* = 26) from patients was performed using an Agilent 7890A gas chromatography system coupled to an Agilent 5975C inert XL EI/CI MSD system (Agilent, Santa Clara, CA, United States). A HP-5MS fused-silica capillary column (30 m × 250 μm × 0.25 μm; Agilent J & W Scientific, Folsom, CA, United States) was used. Sample preparation and GC/MS methods were performed as detailed in the paper of Gao et al. (2009). Peaks obtained from fecal water samples were aligned, grouped and corrected according to the mass to charge ratio (m/z) and retention time using XCMS R tools (Smith et al., 2006). Resulting MS data was a data matrix in which 835 features were found and characterized by a retention time (RT), mass to charge ratio (m/z) and their corresponding intensities per patient. Spectra were deconvoluted using Automated Mass Spectral Deconvolution and Identification System (AMDIS) before being compared with reference libraries (in-house and NIST05). Annotation and identification of features were based on standards proposed by Sumner et al. (2007). Eighty-five features were found statistically relevant and identified based on the retention time, m/z and reference spectra of standards from a local database. Features were considered as putatively annotated when only the m/z and RT and main fragments were matched with the reference standards. The remaining features were putatively characterized (m/z and RT were verified with metabolites in our local database) or noted as unknown features.

### Inputs From Host, Lifestyle, and Microbiota for Partial Least Squares Regression Analysis

**Figure 1:**
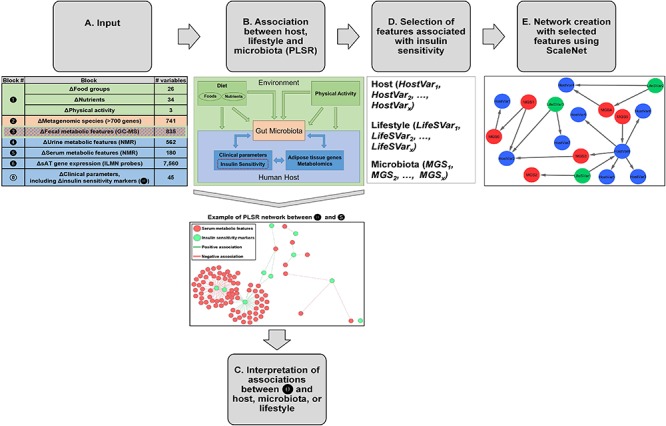
Analysis pipeline for data integration. **(A)** Groups of variables from host, gut microbiota, and lifestyle were considered as input for this analysis. Specifically, the inputs were changes (week 6 – baseline) in the following blocks of variables: 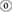 clinical parameters (*N* = 45), including 10 markers of IS/resistance (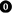), 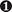 lifestyle factors (food groups, *N* = 26, nutrients *N* = 34, and physical activity, *N* = 3), 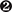 MGS (*N* = 741, i.e., with more than 700 genes), 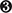 fecal metabolic features (*N* = 835), 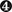 urine metabolic features (*N* = 562), 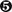 serum metabolic features (*N* = 180), and 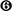 sAT gene expression (*N* = 7,560 ILMN probes). **(B)** Two blocks of variables at a time were analyzed using PLSR with canonical mode (MixOmics R package). We have analyzed changes in 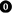 versus changes in 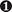 through 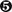, and between 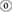 and 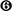. Association coefficient threshold = | 0.7| was selected for analysis with 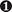 and 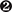, and | 0.75| for analysis with 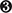, 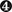, 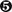 and 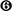. An example of a PLSR network between blocks 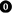 and 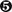 is shown. **(C)** The associations between 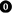 and host, microbiota, or lifestyle were interpreted. **(D)** From the PLSR output, the features most strongly associated with improvement in IS after CR were selected (top 20% variables from each PLSR). **(E)** Visualization of selected features in network reconstruction using SCS (ScaleNet, developed by our group). In hypothetical network shown in E, **red nodes**: microbiota; **blue nodes**: host; **green nodes**: lifestyle factors. Arrows refer to dependency directionality.

A large set of variables measured from elements in host, lifestyle patterns or microbiota have been included in the data integration analysis presented herein. Specifically, as shown in Figure 1A, the following numbers of variables have been included: 26 food groups and 34 nutrients derived from 7-day food diaries, three physical activity variables obtained from the validated Baecke questionnaire, 741 MGS derived from metagenomic sequencing, 835 features from fecal metabolic features obtained using GC–MS, 562 urine and 180 serum metabolic features measured with NMR, 45 clinical parameters (including anthropometric measures, markers of IS, circulating concentrations of lipids, inflammatory markers, adipokines, creatine, and cystatin C, and adipocyte diameter measured from sAT biopsies).

### Partial Least Squares Regression With Canonical Mode

Multivariate methods such as PLSR are well-suited and often applied to big biological data, including situations where the number of variables (clinical parameters, metabolic features, genes, etc.) is much larger than the number of observations (patients). In particular, PLSR is used to model relationships between two groups of variables (Esposito Vinzi et al., 2010). It maximizes the covariance between latent variables which are linear combinations of the observed pairs of variable groups. The results can be efficiently visualized as graphs that enable better understanding of underlying biological relations and processes. In our analysis, we have used the R “mixOmics” package (Omics Data Integration Project), and the function pls(), with the mode = “canonical,” which assumes no directional relationships between data sets. PLSR usually refers to an asymmetric deflation of the two groups of variables, if the deflation is symmetric, then the mode is called canonical mode. A threshold between | 0.7| and | 0.75| for association coefficients was set in order to only consider the strongest connections between each variable block and IS. Networks from PLSR were generated using Cytoscape (Shannon et al., 2003).

### Spectral Consensus Strategy for Accurate Reconstruction of Large Biological Networks

Developing statistical methods to accurately reconstruct biological interaction networks is a central goal of integrative systems biology (Herrgård et al., 2008). In the networks that are of interest in our analysis, nodes represent variables and edges indicate statistical dependency. While many network reconstruction methods exhibit competitive results on various types of data, most state-of-the art methods are challenged to scale to very high dimension datasets where the number of variables might be several orders of magnitude larger than the number of observations. In our analysis, this is precisely the setting we encounter as the number of variables is 9,986 and the number of observations is 27.

To accurately reconstruct such a network of interactions we have developed an original method (Affeldt et al., 2016) that first reduces the reconstruction problem into a large number of much simpler reconstruction problems, then let the lower-dimension problem be solved by state-of-the art reconstruction methods and finally adopt a consensual voting strategy between these methods to identify the most accurate sub-graphs. The different sub-graphs are then connected when they share common nodes like pieces of a larger puzzle. The main originality of the method lies in its powerful problem reduction that, thanks to a so-called spectral decomposition, both identifies non-exclusive sets of most likely dependent variables and enables the approach to scale-up to large biological networks. ScaleNet networks were generated using Cytoscape (Shannon et al., 2003). For the network presented herein, we used 14% of the eigenvector, and the size of the subgraphs used for the reconstruction was 0.03.

### Prediction and Regression

The target variables being predicted (i.e., revised QUICKI index and HOMA-B as markers of IS) are continuous, therefore, we performed a linear regression to estimate the impact of each set of independent of variables on the variable of interest. The MSE provides the average of the squares of the errors of deviations, and quantifies the difference between the real and estimated data. To measure the impact of each data source on IS, we ran linear regressions and computed the MSE for each type of data (10-fold cross validation) (Hastie et al., 2009). We inferred that the inverse values of the MSE represent the magnitude of the impact. The intuition behind such a visualization is that the lower the error, the more significant the data are.

### Analysis of Subcutaneous Adipose Tissue *DDRGK1* in a Separate Obese Population

To explore some of the novel links identified with PLSR and ScaleNet, we used existing data from an unpublished CR study and determined association between sAT *DDRGK1* and IS. The methodologies for these variables were the same that in the present study. The validation study consisted of a 6-week very low calorie diet, followed by 6 weeks of weight stabilization. In this group of overweight/obese adults, only baseline and week 12 values (i.e., after the weight regain and stabilization period) are presented.

### Availability of Data

Data used in this manuscript can be accessed as follows: (1) AT Illumina microarray data with Gene Expression Omnibus (GSE112307), (2) serum metabolomics NMR data with Metabolights (pending accession code), (3) urine metabolomics NMR data with Metabolights (pending accession code), (4) fecal metabolomics GC–MS data with Metabolights (MTBLS653), and (5) metagenomics raw solid read data with the European Bioinformatics Institute European Nucleotide Archive (ERP003699).

## Results

### Dietary Intervention-Induced Weight Loss and Improved Insulin Sensitivity: Multiple Inputs Used for Data Analysis and Integration

Figure 1 shows an overview of the different steps taken to analyze the data presented in this study. Details are provided in Section “Materials and Methods.” The study population is a subgroup from the MICRO-Obes study (Cotillard et al., 2013; Kong et al., 2013, 2014), consisting of 27 overweight or obese adults who underwent a 6-week CR intervention (1,200 kcal/day for women and 1,500 kcal/day for men). The diet was low in fat (25% of energy intake), high in protein (35% of energy intake), and rich in total fiber and in carbohydrates with a low glycemic index. The diet composition has been described elsewhere (Rizkalla et al., 2012). The variable inputs as described previously (Cotillard et al., 2013; Kong et al., 2013, 2014), were divided into the following blocks (Figure 1A): 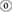 45 clinical variables that included anthropometry, adipocyte diameter, measures from blood chemistry profiles (including 10 IS markers, **
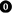
**), and inflammatory markers; 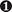 lifestyle factors (26 food groups, 34 macro- and micronutrients, 3 indexes representing degree of physical activity at work, sports and leisure time); 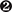 741 MGS; 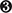 835 fecal metabolic features; 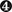 562 urine metabolic features; 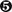 180 serum metabolic features; and 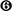 7,560 genes expressed in sAT. The change (week 6 – baseline) in each variable was exclusively used in all analytical steps (Figure 1B–E).

As expected, the dietary intervention led to a significant reduction in BMI, with an average weight loss of 5.4 ± 2.5%, as well as waist-to-hip ratio, % fat mass and android fat (Table 1). There was a significant decrease in blood lipids, and a marked improvement in surrogate markers of insulin secretion (HOMA-B, calculated as described in Levy et al., 1998); or IS (HOMA of insulin resistance, HOMA-IR, HOMA-S, Disse index, the quantitative IS check index, QUICKI, the revised QUICKI, insulin to glucose ratio, and the new FIRI, Table 1). It is important to note that there was a wide range of change in IS, with both responders and non-responders to the intervention, as is commonly the case with lifestyle and CR interventions (Kong et al., 2013). Indeed, while all participants experienced a decrease in BMI during this period, there was individual variability in change of the revised QUICKI, with 19 individuals having an increase (improving IS as measured by this index) and 8 individuals having a decrease in this index. As such there was no significant improvement in mean revised QUICKI (Table 1). Supplementary Figure 2 shows individual trajectories for IS indexes used in the analysis.

**Table 1:** Change in clinical outcomes with calorie restriction.

		Baseline		Week 6		
Variable	Number	Median	IQR	Median	IQR	P_adj_
Age	27	41	26			
Sex M	3					
*F*	24					
BMI (kg/m^2^)	27	33.9	5.8	31.9	5.9	**1.3E-08**
Hip circumference (cm)	27	118	12	114	15	**4.17E-08**
Waist circumference (cm)	27	107	14	99	13	**7.4E-07**
Waist-to-Hip ratio	27	0.92	0.12	0.89	0.13	**0.0217**
Fat mass (%)	27	42.7	6.9	41.6	6.1	**0.0002**
Fat free mass (%)	27	54.3	6.6	55.5	6.2	**0.0005**
Android fat (%)	27	59.4	8.3	57.7	9.5	**0.0117**
Gynoid fat (%)	27	38.5	7.8	38.6	9.8	**0.0383**
Adipocyte diameter (μm)	27	110.9	11.5	101.7	6.1	**1.3E-08**
Serum triglycerides (mM)	27	1.08	0.63	0.82	0.53	**0.0010**
NEFA (mM)	27	0.49	0.29	0.50	0.32	**0.0485**
Cholesterol (mM)	27	5.21	1.27	4.66	1.03	**4.63E-05**
HDL (mM)	27	1.35	0.50	1.11	0.41	**0.0002**
LDL (mM)	27	3.30	0.99	2.97	0.94	**0.0016**
CRP (mg/L)	27	3.60	5.25	3.12	4.17	0.40
IL-6 (pg/ml)	27	1.24	1.48	1.47	1.10	0.97
Insulin (μUI/ml)	27	8.6	5.1	5.7	3.7	**1.3E-08**
Glucose (mM)	27	5.1	0.5	5.0	0.4	**0.0032**
Insulin : Glucose	27	1.75	0.90	1.27	0.48	**0.0005**
HOMA2-IR	27	1.15	0.71	0.74	0.48	**1.3E-08**
HOMA2-B	27	94.0	30.9	78.3	32.9	**5.06E-05**
HOMA2-S	27	87.1	68.2	135.2	83.5	**1.67E-08**
FIRI	27	1.93	1.19	1.12	0.77	**1.3E-08**
Disse Index	27	-6.7	4.5	-4.1	6.8	0.06
QUICKI	27	0.34	0.04	0.37	0.04	**1.3E-08**
Revised QUICKI	27	0.39	0.06	0.41	0.05	0.12

*Wilcoxon signed rank sum test, adjusted *P*-values (Padj, BH correction) are shown. BMI, body mass index; NEFA, non-esterified fatty acids; HDL, high density lipoprotein; LDL, low density lipoprotein; CRP, C-reactive protein; IL-6, interleukin-6; FIRI, new fasting insulin resistance index; revised QUICKI, revised quantitative insulin sensitivity check index; IQR, interquartile range. Bold values are significant adjusted *p*-values*.

### Change in Insulin Sensitivity After Diet-Induced Calorie Restriction: Association With a Myriad of Lifestyle, Gut Microbiota, Metabolic Features, and Adipose Tissue Genes

Using PLSR with canonical mode, we examined association networks between the changes in IS from this CR intervention and changes in lifestyle factors (i.e., diet and physical activity), blood, urine, and fecal metabolomics, gut metagenomics, and sAT transcriptomics. Although there is redundancy in some of the IS markers, they were included to ensure analytical relevance. In other words, we would expect similar indexes such as FIRI and HOMA-IR to be associated with consistent sets of variables (which was the case, as seen in Supplementary Figure 3).

**Figure 2:**
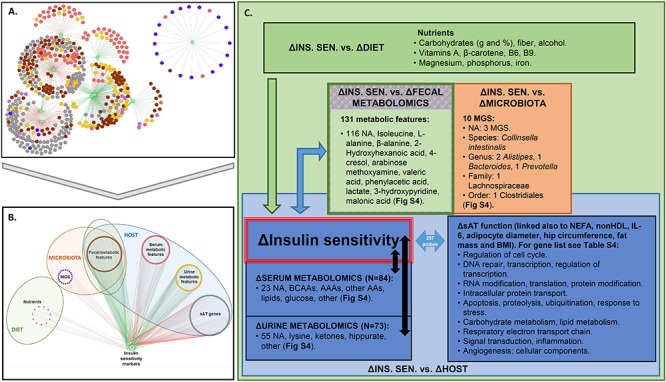
Association between changes in IS and factors in host, microbiota, and lifestyle. **(A,B)** Superimposed PLSR networks associated with change in insulin sensitivity (ΔINS. SEN.), where nodes are arranged by **(A)** betweenness centrality and **(B)** variable type. The **green edges** correspond to positive correlations of change and the **red edges** correspond to negative correlations of change. **(C)** Summary of variables from host, microbiota, and lifestyle factors associated with ΔINS. SEN. Association coefficient threshold = [0.7] for lifestyle factors, and | 0.75| for metabolomics and sAT gene expression. NA, not annotated; BCAAs, branched chain amino acids; AAAs, aromatic amino acids; AAs, amino acids; MGS, metagenomic species. No association with change in physical activity or food groups was found above the selected threshold.

The networks depicting PLSR results (Figure 2A,B) show strong associations of change between several groups of variables and IS markers. We considered association coefficients greater than | 0.7| or, in some cases, | 0.75|. These thresholds represent a trade-off between analytical stringency and retention of useful information (Supplementary Table 2). Above the selected thresholds, there were associations between improvements in IS and change in nutrient intake that included fiber, carbohydrate, alcohol, β-carotene, vitamins A, B6, and B9, magnesium, phosphorus, and iron (Figure 2C and Supplementary Figure 4A, Supplementary Table 3). No food groups were associated with IS above the selected threshold.

Regarding gut microbiota changes, 10 MGS, each detected in at least two subjects, were associated (in some cases negatively and in others positively) with improvement in IS after CR. Three of these MGS have not been annotated at any taxonomic level (Figure 2C). Of the annotated ones, when considering the lowest taxonomic annotation available, one was related to the species *Collinsella intestinalis* (having negative association of change with revised QUICKI). At the genus level there were two *Alistipes* (having positive association of change with revised QUICKI, Disse index or glucose), one *Bacteroides* and one *Prevotella* (both having positive association of change with glucose). At the family level one Lachnospiraceae (having negative association of change with glucose); and at the order level one Clostridiales (having negative association of change with revised QUICKI) (Supplementary Figure 4C). Of the unannotated MGS, there was one having a negative association of change with the Disse index (Best Hit: uncultured *Faecalibacterium* sp.), and two having a negative association of change with fasting glucose (Best Hit: *Opitutaceae bacterium* TAV5 and uncultured *Faecalibacterium* sp.).

There were 84 serum metabolic features whose changes in concentration were most strongly associated (81 negatively and 3 positively) with improvement in IS. Among these were BCAA and AAAs, as well as other amino acids, namely lysine, glutamate, and glutamine (Figure 2C and Supplementary Figure 4B). IS improvement was associated with a decrease in BCAA, consistent with the literature (Roberts et al., 2014). Several explanations could account for the implication of BCAA in glucose homeostasis. One hypothesis is the potential role of AT, which has been shown to have decreased expression of BCAA catabolic enzymes in obesity and insulin resistance (Roberts et al., 2014). In agreement with this hypothesis, the PLSR analysis between variation in serum metabolic features and sAT transcriptomics show strong significant associations between sAT genes and BCAA as well as with other metabolites including 3-hydroxyisobutyrate and 3 methyl-2-oxovalerate (Supplementary Figure 5). When considering change in AT genes coding for enzymes involved in BCAA catabolism (Supplementary Figure 5F) (Herman et al., 2010), there was a positive association of change between valine and isoleucine with the *BCAT2* gene. This enzyme converts BCAA into α-ketoacids in the first step of BCAA catabolism (Herman et al., 2010). There was a negative association between all BCAA and gene expression of *DBT*, which is part of the BCKDH complex. This complex catalyzes the second step of BCAA catabolism, namely the conversion of α-ketoacids into acyl-CoA esters. Only isoleucine was positively associated with *BCKDHA*, which is also part of the BCKDH complex. *ALDH6A1*, involved in a downstream step of the pathway, was also negatively associated with variations in all BCAA. These results suggest that while substrates to the first step of BCAA catabolism may decrease with weight loss, subsequent steps of the pathway increase, reflecting an upregulation of BCAA catabolism.

In urine, change in hippurate, a metabolite previously found to be inversely associated with metabolic syndrome risk, gut microbiota richness (Pallister et al., 2017), and obesity (Elliott et al., 2015), was positively associated with improvement in IS, specifically, with a decrease in plasma insulin, HOMA-IR and FIRI (Supplementary Figure 4B). Fecal metabolites associated with change in IS included isoleucine, alanine, β-alanine, 2-hydroxyhexanoic acid, arabinose methoxyamine, valeric acid, phenylacetic acid, 3-hydroxypyridine, 4-cresol, malonic acid, and lactate (Supplementary Figure 4B).

PLSR analysis also revealed that the clinical factors most strongly associated with AT genes were Disse, QUICKI, FIRI and revised QUICKI indexes, NEFAs, non-HDL, adipocyte diameter, interleukin-6 (IL-6), BMI, hip circumference and fat mass (in kg) (Supplementary Figure 4D). There were 257 genes associated with these clinical factors (summarized in Figure 2C and listed in Supplementary Table 4).

In summary, this PLSR analysis, where we have applied stringent association coefficient thresholds, has resulted in the identification of a large set of factors (*N* = 565) from multiple sources that are connected to changes in IS after CR.

### Data Reduction and Integration: Novel Connections Between Insulin Sensitivity, BCAA and *DDRGK1*, an Adipocyte Expressed Gene Involved in Protein Ubiquitination

Our next objective was to gain a deeper insight into these large sets of interactions between variables and highlight novel connections based on the strongest associations between in CR-induced changes in IS, metabolic features, sAT genes and MGS. To reduce the number of features, we examined the top 20% associations which was a trade-off between stringency and retention of strong associations with change in IS (Supplementary Table 5).

We used ScaleNet, a network learning approach based on SCS, developed by our group (Affeldt et al., 2016), to examine and visualize the strongest statistical dependencies between CR-induced IS changes and the selected variables. The data reduction approach led to focus on the following features: three nutrients (carbohydrate in % of energy intake, fiber in grams, and alcohol in grams), MGS (three species with highest prevalence in subjects, namely GU:41 from the Clostridiales order, GU:84 from the *Alistipes* genus, and GU:228 which is not annotated but whose strongest hit from the gene catalog is *Faecalibacterium* sp.), 22 serum metabolic features, 19 urine metabolic features, 24 fecal metabolic features, the markers of IS, adipocyte diameter, NEFA, and 32 sAT genes.

ScaleNet thus established statistical dependencies between these variables based on the consensus taken from different network reconstruction approaches. The dependencies derived through ScaleNet are therefore more robust than if any one single network reconstruction method were used. These statistical dependencies represent the pairwise mutual information between variables, and so they capture both linear and non-linear relationships.

**Figure 3:**
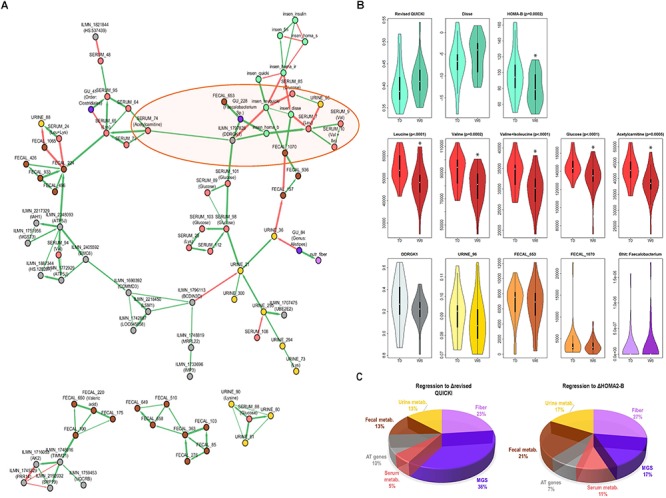
Connection between change in IS, BCAAs and other factors from host, lifestyle, and microbiota. **(A)** ScaleNet network reconstruction showing how changes in variables most strongly associated with improvements in IS are associated with each other. The **green edges** correspond to positive correlations of change and the **red edges** correspond to negative correlations of change. **(B)** Change from baseline to week 6 in select variables from network (highlighted in **orange ellipse** on the network in **A**). When significant, adjusted *P*-values (BH correction) from Wilcoxon Signed Rank test are shown. **(C)** Linear regression model, where each section of the pie chart shows the relative contribution of change in selected variables (grouped by class) to change in revised QUICKI and HOMA-B. Variables included in the model were those found in the main cluster of the network in part A (except serum metabolic features annotated as glucose). MGS, metagenomic species; metab., metabolic features; *DDRGK1*, DDRGK domain containing 1 (also known as *Dashurin*).

As shown in Figure 3A, the reconstructed network is composed of a large cluster (top) and several smaller clusters not connected to the large cluster (bottom). This network showed several within-class modules, such as for fecal metabolic features for which a distinct cluster of nodes (variables) can be seen, sAT genes, or serum metabolic features, where several features corresponding to serum glucose clustered together. Importantly, we observed statistical dependencies depicting already established connections between classes of variables (Figure 3A). Indeed, the IS markers that clustered together were strongly connected with a serum BCAA cluster (leucine, valine, and valine + isoleucine).

This network showed a novel direct connection between the sAT gene *DDRGK1* (also known as UFM1-Binding Protein 1, or *UFBP1* or *Dashurin*) and three markers of IS (glucose, HOMA-B, and revised QUICKI), as well as with serum acetylcarnitine and GU:228 (unannotated, with closest relevance to *Faecalibacterium* sp.), and an indirect connection with other markers of IS and serum BCAA. The *DDRGK1* gene has not been previously explored in human AT. Revisiting existing “in house” microarray data from obese women (unpublished) revealed that *DDRGK1* is 1.6-fold more highly expressed in isolated adipocytes than stromal vascular fraction in sAT (Supplementary Figure 6). To further examine the relevance of this relationship between *DDRGK1* and IS variation, we explored another group of 27 overweight and obese patients with comparable clinical characteristics (unpublished data, Supplementary Table 6). Here, the subjects had undergone a very low-calorie diet for 6 weeks followed by a 6-week weight maintenance period, and *DDRGK1* expression was examined after 12 weeks (Supplementary Figure 7). After this weight maintenance phase, there was a significant positive correlation of change between the revised QUICKI and *DDRGK1* expression in sAT (Supplementary Figure 7C).

The changes in selected variables having dependencies with the IS cluster is displayed in Figure 3B, showing a significant decrease in serum BCAA and acetylcarnitine, a tendency to decrease in *DDRGK1*, and no significant change in GU:228, or unannotated urine and fecal metabolic features directly connected with the IS cluster. The network also showed dependencies between serum lysine and different elements including blood glucose, unannotated fecal metabolic features, an MGS from the Clostridiales order, and acetylcarnitine.

### Insulin Sensitivity Improvement After CR: Important Contribution of Metagenomic Species and Fiber Consumption in the Diet

The ScaleNet network showing the statistical dependencies of highly connected variables (Figure 3A) suggests that not only lifestyle factors, fecal, urine, and serum metabolic features, but also AT genes and gut microbiota composition contribute to improvements in IS after CR. We then examined the relative contribution of these elements to improved IS after CR. We used linear regression and computed the inverse of the MSE by variable class.

The pie charts on Figure 3C show that the greatest contribution (around 50%) to improvement in revised QUICKI and HOMA-B index came from change in abundance of the three MGS included in the network, increase in dietary fibers and, in the case of HOMA-B, change in concentration of fecal metabolic features. Interestingly, there were similar contributions from sAT gene expression (including *DDRGK1*) and selected serum and urine metabolic features (between 5 and 17%). These results also suggest important contributions by gut microbiota MGS and dietary intake of fiber to improvements in IS.

## Discussion

In this study, we have identified novel links between host glucose homeostasis, lifestyle factors and gut microbiota in the context of a 6-week CR intervention. This is to our knowledge the first study integrating information from lifestyle factors, gut microbiota composition, clinical factors, sAT gene expression, and metabolomics from serum, urine and feces, to investigate their relationship with IS changes after CR. The variables that were found to be more strongly associated with changes in IS should be explored in future studies as potential biomarkers or predictors of individual responses to this kind of intervention.

The analytical approach presented herein has led to: (1) integration and visualization of multiple associations between changes in gut microbiota, human omics data, lifestyle factors, and IS; (2) reduction of data dimensionality to highlight key variables of interest (from 9,986 variables to 115 variables included in the ScaleNet reconstruction); (3) identification of a robust inverse association between changes in circulating BCAA with IS during CR; and (4) identification of new links between IS and different elements such as those seen with metabolic features in urine and feces, sAT genes and gut microbiota composition. (5) Finally, we show an important contribution of MGS in improved IS after CR in link with diet modulation.

### Decreased Serum BCAA Associated With Increased Insulin Sensitivity Are Connected With Features From Host, Lifestyle, and Gut Microbiota

One of the strongest associations with IS improvement was a significant decrease in BCAA with CR (Figure 3B). This differs from previous findings comparing metabolomics profiles between a gastric bypass intervention and a weight-matched dietary intervention (Laferrère et al., 2011). The authors observed a decrease in BCAA and their metabolites in the surgical group, concomitant with amelioration in IS. However, this was not the case in the dietary intervention group. The baseline BMI for this group was considerably higher than in our study group (42.8 ± 3.8 vs. 34.0 ± 4.1 kg/m^2^, respectively), and their insulin resistance markers remained higher than what we have observed in this study after the dietary intervention. Therefore, greater weight loss may have been necessary to yield significant decrease in BCAA related to an improvement in IS, as shown in the present study. Associations were identified not only between BCAA and IS, but also with sAT gene expression, corroborating the hypothesis of a potential role of sAT in insulin resistance through inhibition of BCAA catabolism in AT which may accompany the known alteration of other metabolic pathways, including mTOR signaling in skeletal muscle, increase in gluconeogenesis, decrease in glycolysis and β-oxidation, and increased mitochondrial stress (Newgard et al., 2009; Herman et al., 2010; Roberts et al., 2014).

Our data integration and reduction approach led to the identification of robust dependencies between the IS markers/BCAA cluster in the ScaleNet network with *DDRGK1*, a gene involved in protein ubiquitination. The relationship between *DDRGK1* and IS was confirmed in an independent group of overweight/obese subjects following a different dietary intervention setting. Indeed, this second group was explored during a weight maintenance phase after a period of weight loss. Thus the fact that AT gene expression profiles greatly vary depending on the stage of weight loss and regain (Capel et al., 2009; Piening et al., 2018) could account for the difference in the direction of correlation between this study population and the independent clinical group.

*DDRGK1* has been shown to become activated and bind to *UFM1* in pancreatic beta cell lines for protection against ER stress-induced apoptosis (Lemaire et al., 2011). There is evidence in cell lines that DDRGK1 regulates NF-κB through interaction with IκBα (Xi et al., 2013). Limited evidence is available about this gene’s function in AT. We show here that it is more highly expressed in isolated adipocytes than in the stromal vascular fraction calling for deeper exploration of this gene in adipocyte metabolic pathways. The ScaleNet network also shows a link between *DDRGK1* and an unannotated MGS whose strongest alignment is with *Faecalibacterium* sp. Interestingly, this microbial genus has been shown to be anti-inflammatory and associated with IS (Sokol et al., 2008; Le Chatelier et al., 2013; Munukka et al., 2014).

The network also showed elements from sAT, microbiota composition and fecal and urine metabolomics connected to the IS and BCAA cluster. Acylcarnitines are intermediates of fatty acid oxidation that, when elevated, may be involved in lipid-induced pathways of insulin resistance, although this hypothesis has been questioned (Schooneman et al., 2013). Studies have conversely shown that acetyl-L-carnitine supplementation leads to improved IS in insulin-resistant overweight and obese individuals (Ruggenenti et al., 2009). Acylcarnitines may also derive from other metabolites, including ketone bodies, BCAA, lysine and, in the case of acetylcarnitine, glucose. Acetylcarnitine, the two-carbon acylcarnitine, may be an indicator of glucose and fatty acid catabolism status and has been previously associated with insulin resistance (Schooneman et al., 2013). A recent weight loss study in obese subjects showed that acylcarnitines, including acetylcarnitine, were positively correlated with NEFAs at baseline (Schooneman et al., 2016). In this study, contrary to our findings and other published evidence (Ruggenenti et al., 2009; Muoio et al., 2012), acetylcarnitine increased after a weight loss intervention and was not correlated with IS. The design of this study differed from ours in that it was a 12-week intervention including a group on diet with physical activity and another on pharmacological treatment. The differences in study design (including diet) and effects induced on host metabolism from these interventions could explain the discrepancies between the studies. Here, concentration of acetylcarnitine decreased significantly, and on the ScaleNet network (Figure 3A) it was directly connected with *DDRGK1*, and indirectly connected with BCAA, IS markers and a MGS from the Clostridiales order (GU: 41). This suggests a possible link between ER stress in sAT, and insulin resistance-associated dysregulation of AT metabolism. This hypothesis calls for confirmation in future studies, both involving large human cohorts, as well as mechanistic exploration in *in vivo* and *in vitro* models.

### Cross-Talk Between Gut Microbiota Composition and Insulin Sensitivity After CR

The cross-talk between microbiota and metabolism in various organs and tissues, including AT, has been described mostly in mice but less so in humans (Tremaroli and Bäckhed, 2012; Geurts et al., 2014; Fetissov, 2017). Even though we focused our study on peripheral organs, there is current interest in the cross-talk between gut microbiota and brain signaling (Wang et al., 2018). Gut microbiota likely has a role in BCAA-associated development of insulin resistance, as shown by Pedersen et al. (2016), where a connection between circulating BCAA, increased BCAA synthesis and decreased BCAA import in microbiota was identified in insulin resistant adults. In the same study, a causal role for gut microbiota in increase of circulating BCAA and insulin resistance was suggested in a mouse model. Consistently, our results demonstrate that a reduction of caloric intake, body weight and improvement in dietary quality results in a significant decrease in BCAA, which is connected with improvement in IS and potentially also sAT metabolism and protein ubiquitination, and with compositional changes in the gut microbiota. The relative contribution to circulating BCAA, as well as other metabolites, from the gut microbiome and host and the degree of gut permeability before and after weight loss interventions in human obesity are to be considered in future studies (Genser et al., 2018).

### Challenges in Integration of Data With High Dimensionality

A limitation of this study is the small sample size, an issue generally encountered in studies using big data and deep phenotyping. To circumvent this issue, we have used stringent approaches in the different steps of the analysis, including high thresholds for selection of relevant variables and methods adapted to high dimensionality such as the local reconstruction aspect of ScaleNet. The advantage of this method is that it goes far beyond pairwise associations of variables, taking into account all long-range associations within subsets of variables, considering both linear and non-linear relationships. This method is adapted to high-dimensional settings since each reconstruction method uses many local reconstructions based on a spectral setting. However, it is important to note that the links we have identified need to be corroborated by future research, including comparable study designs and similar population characteristics. In fact, this analytical approach will be applied to the Metacardis study population^1^.

This study included only three men, which prevented the assessment of sex differences in our outcomes. Future studies should be balanced for gender to identify potential sex-specific effects relevant in the treatment of obesity. Another limitation is that some metabolic features included in this analysis could not be annotated due to lack of knowledge in public databases and/or limitations in the identification of low intensity metabolites, so interpretation of these results could evolve depending on the depth of our abilities to annotate further features.

The PLSR analysis takes into account pairwise associations between two sets of variables rather than all possible connections between and within both groups of variables. Our approach started with a pairwise assessment of connections with IS in order to prioritize variables of greatest relevance, followed by the ScaleNet analysis, which included all possible connections between these variables. However, the fact that we have initially considered associations between two groups of variables at a time (i.e., IS markers versus each block of variables), instead of taking a more global approach, may have masked other important connections between variables. Conversely, the approach we have used makes the results more interpretable.

## Conclusion

Our data integration approach has provided new insights and revealed new associations between IS and AT genes, metabolites, lifestyle factors, and microbiota during CR. Although here we present some initial steps toward validation of our observations, this approach should be repeated in other populations with similar characteristics, and ideally with larger sample sizes, to determine the robustness and reproducibility of the more novel links we have identified. A recent study has also taken a data integration approach to study multi-omics associations during weight gain and weight loss, and have uncovered connections between biomarkers and the gut microbiota in individuals undergoing weight gain followed by weight loss (Piening et al., 2018). Similarly to our study, Piening et al. (2018) highlight connections between IS status and circulating BCAA and acylcarnitines. It is through reproducing such strategies in different studies that a reliable and more complete picture of the connection between obesity, metabolism, gut microbiota, and lifestyle factors will be obtained.

This type of integrative approach should lead to innovative applications for personalized care. Individual response profiles from weight loss interventions could be optimized by targeting markers of interest identified through this analysis. Data integration has the potential for the development of personalized approaches that would allow us to tackle obesity and other associated cardiometabolic diseases.

## Author Contributions

MD, SR, J-DZ, and KC have designed the overall study. KC and SR have designed and conducted the clinical research and clinical data management. MD managed analysis and interpretation of results. MD, NS, RB, SA, EP, J-DZ, and KC developed the analysis strategy. JA-W and BK contributed to clinical data analysis. VP carried out adipose tissue gene expression analysis and validation. BH and EV contributed to dietary data analysis. M-ED, JC, and LH carried out the serum and urine metabolomics analysis. FI, EP-G, and CJ carried out the fecal metabolomics analysis. EP, JD, and J-DZ conducted the microbial data analysis. All authors contributed to the preparation, writing, and approval of the manuscript.

## Conflict of Interest Statement

Co-authors from Danone Nutricia Research are part of the METACARDIS Consortium and they have contributed to the work presented herein in this capacity. The remaining authors declare that the research was conducted in the absence of any commercial or financial relationships that could be construed as a potential conflict of interest.

## Footnotes

1 http://www.metacardis.net/

## Abbreviations

AAAs: aromatic amino acids
*ALDH6A1*: aldehyde dehydrogenase 6 family, member A1
AT: adipose tissue
*BCAT2*: branched chain amino acid; transaminase 2
BCAA: branched chain amino acid
BCKDH complex: branched chain ketoacid dehydrogenase
*BCKDHA*: branched chain ketoacid dehydrogenase E1
BH: Benjamini-Hochberg
BMI: body mass index
CR: calorie restriction
*DBT*: dihydrolipoamide branched chain transacylase E2
*DDRGK1*: DDRGK domain containing 1
FIRI: fasting insulin resistance index
HOMA-B: homeostasis model assessment of beta cell function
HOMA-IR: homeostasis model assessment of insulin resistance
HOMA-S: homeostasis model assessment of insulin sensitivity
IL-6: interleukin-6
IS: insulin sensitivity
MGS: metagenomic species
MSE: mean squared error
NEFAs: non-esterified fatty acids
NF-κB: nuclear factor-κB
non-HDL: non-high density lipoprotein = total cholesterol – HDL
PLSR: partial least squares regression
pSVA: permuted-surrogate variable analysis
QUICKI: quantitative insulin sensitivity check index
revised QUICKI: revised quantitative insulin sensitivity check index
sAT: subcutaneous adipose tissue
SCS: spectral consensus strategy
T2D: type 2 diabetes
*UFM1*: ubiquitin-fold modifier 1
