# Supplementary material for: A Data Integration Multi-Omics Approach to Study Calorie Restriction-Induced Changes in Insulin Sensitivity
Source: Front Physiol. 2019 Feb 5;9:1958. doi: 10.3389/fphys.2018.01958 (PMC6371001; doi:10.3389/fphys.2018.01958)
Supplement: Supplementary file 2 [file Data_Sheet_1.docx]

Supplementary Material

# Supplementary Figures and Tables

## Supplementary Figures


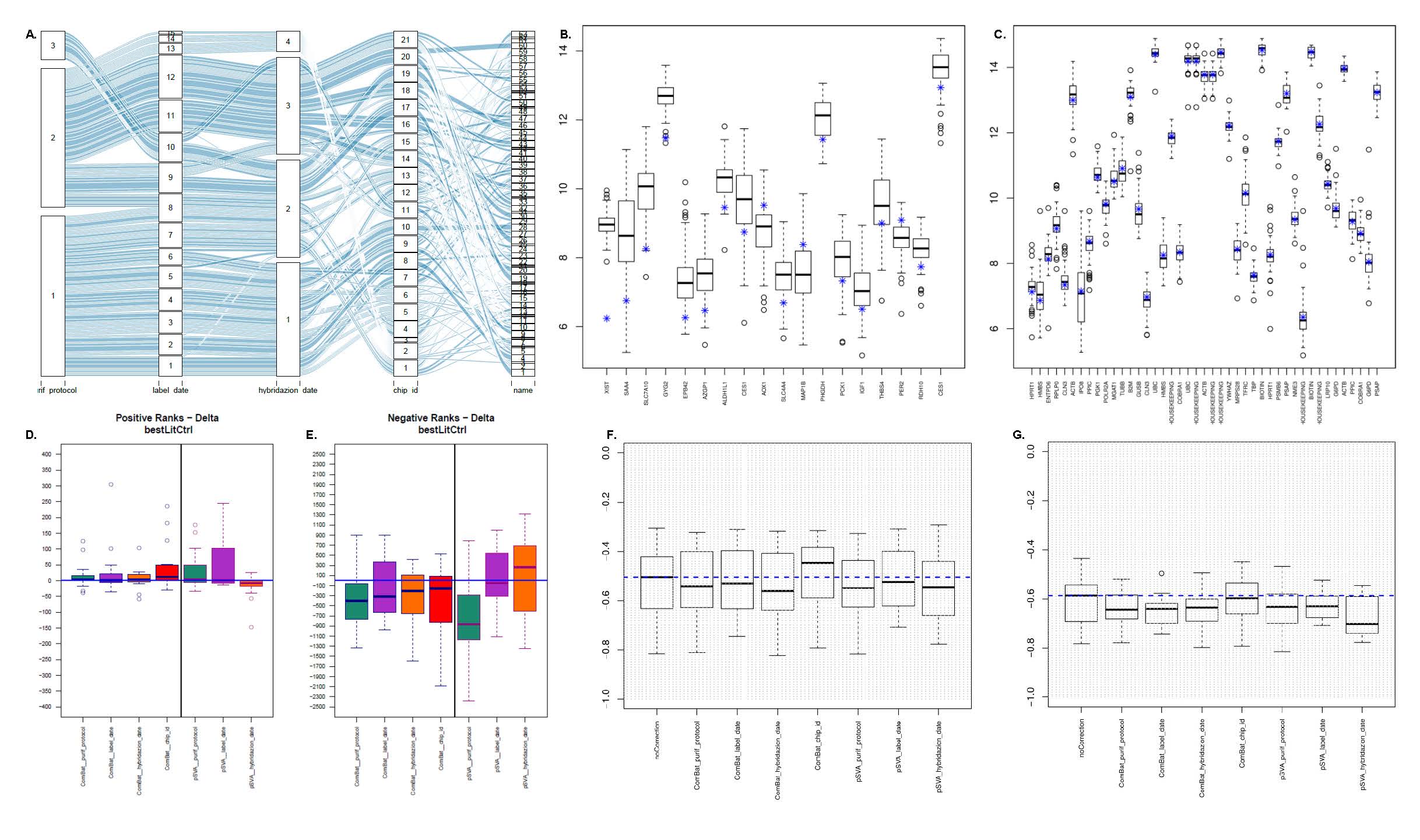


**Supplementary Figure 1. Batch correction of AT gene expression.**

**A.** Alluvial view of the distribution of each ILMN chip sample through the different batch effect variables. Purifying protocols, labelling dates and hybridization dates are temporally ordered. Each line symbolizes a chip sample.

**B.** Positive controls (N=18) identified from the literature and in our laboratory. The boxplot gives the median and variability expression in the female samples. The blue stars provide the median expression in the male samples.

**C.** The best negative controls (N=43) have been identified from the literature and in our laboratory. The boxplot gives the median and variability in expression in the female samples. The blue stars provide the median expression in the male samples.

**D, E.** P-value rank delta. Delta between the rank of the p-values of the best positive (D) and negative (E) controls before and after batch effect correction.

**F, G.** Spearman correlations between gene expression assessed by ΔCt qPCR values and expression data from the microarray analysis at week 6. Among the 64 genes, a p-value lower than 0.1 is obtained for 28 genes (F) and a p-value lower than 0.01 is obtained for 11 genes (G). The ΔCt qPCR values are log scaled. The selected batch correction method was the permuted-Surrogate Variable Analysis (pSVA) approach.


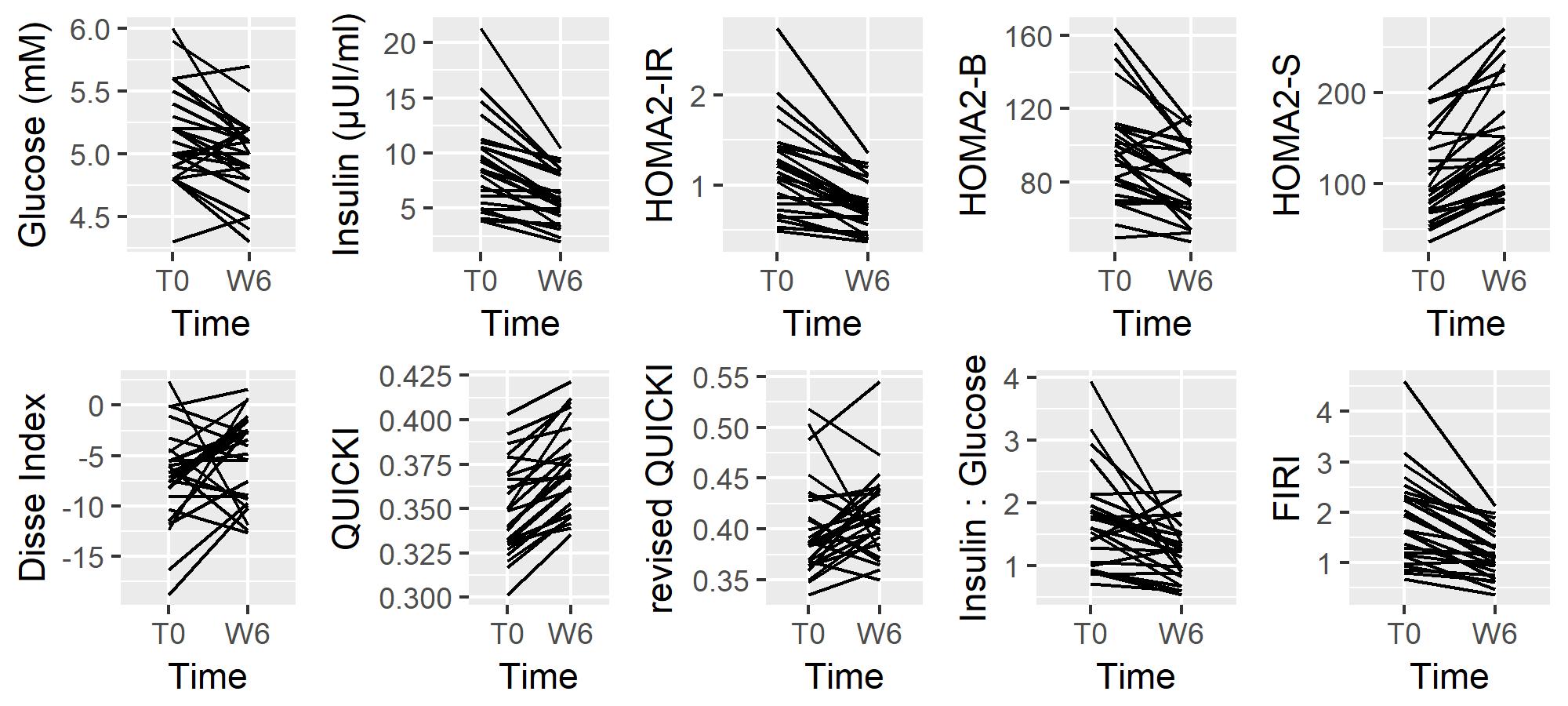


**Supplementary Figure 2. Individual trajectories for insulin sensitivity markers.**

The individual change for the ten insulin sensitivity markers used in this analysis are shown.


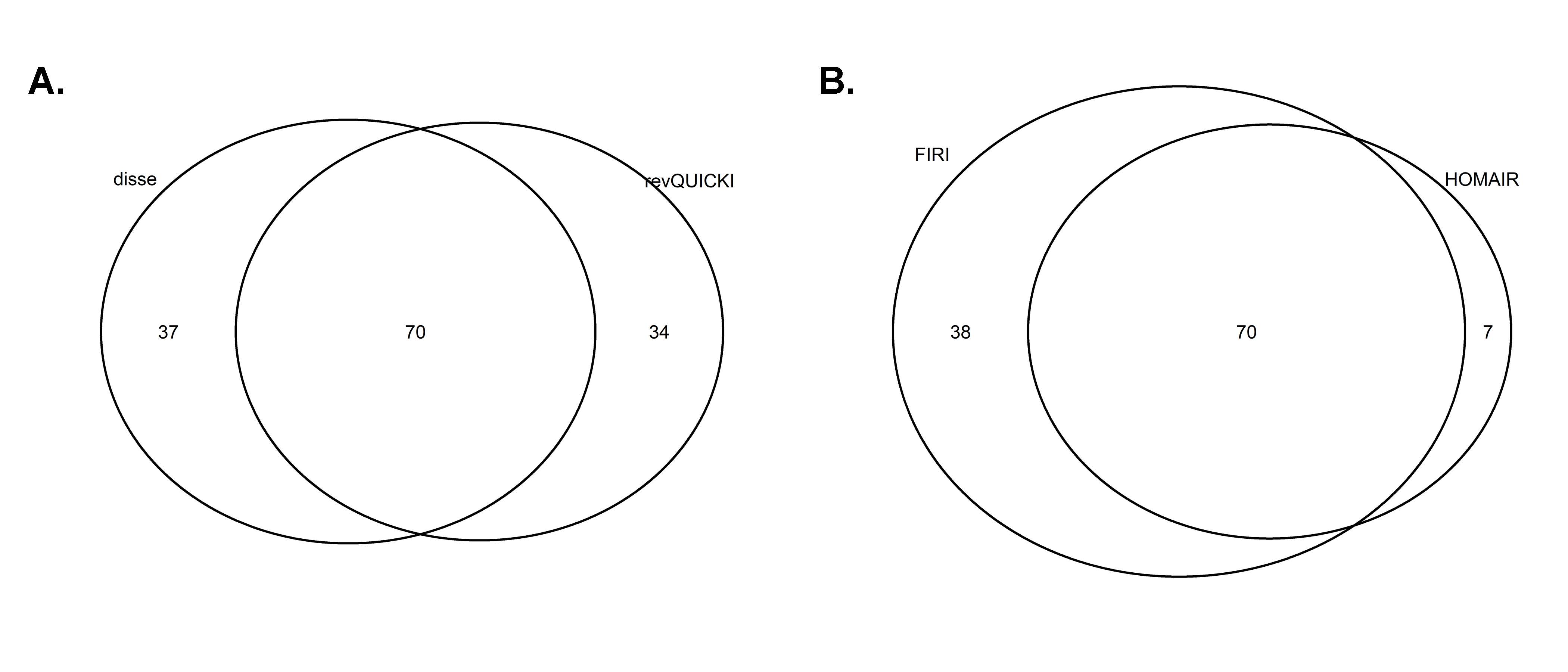


**Supplementary Figure 3. Overlap in association profiles between different insulin sensitivity/resistance markers.**

Numbers on Venn diagrams refer to number of associations between the indicated insulin sensitivity index and other variables from host, microbiota and lifestyle factors.

**A.** Overlap in associations between FIRI and HOMA-IR.

**B.** Disse index and revised QUICKI index. Formulas: HOMA-IR = Based on HOMA2Calculator (Levy *et al*. PMID: 9839117); FIRI = (Glucose x Insulin) / 25; Disse Index = 12 x [2.5 x (HDL / TC) - NEFA] – Insulin; Revised QUICKI = 1 / [log(Insulin) + log(Glucose) + log(NEFA)]; HDL = high density lipoprotein. TC = total cholesterol; NEFA = non-esterified fatty acids; revQUICKI = revised quantitative insulin sensitivity check index; FIRI = New fasting insulin resistance index.





**Supplementary Figure 4. Association between improvement in insulin sensitivity/resistance markers, nutrient intake and metabolomics data.**

Heatmaps showing PLSR association coefficients between change in insulin sensitivity and:

**A.** Change in nutrient intake (PLSR association coefficients above |0.7|).

**B.** Change in metabolic feature concentration (urine, serum, feces; PLSR association coefficients above |0.75|; only associations with annotated metabolites are shown). The numbers of unannotated metabolic features were 23 for serum, 55 for urine, and 116 for feces.

**C.** Change in metagenomic species abundance (species detectable in at least 2 subjects, PLSR association coefficients above |0.70|).

**D.** PLSR analysis between change in clinical factors and change in adipose tissue gene expression (PLSR association coefficients above |0.75|).





**Supplementary Figure 5. Association between changes in adipose tissue gene expression and serum metabolic features.**

**A.** PLSR analysis of association of change between sAT gene expression and serum metabolic features. Only association coefficient above a threshold of |0.8| have been considered.

**B.** Distribution of connectivity degree of nodes seen in A, presented in unmodified scale (top) and log scale (bottom), with red lines indicating 95^th^ percentile of degree distribution (50 connections).

**C.** Network displaying only nodes connected to at least 50 other nodes, their connections to each other, and their annotations: *IGBP1* = immunoglobulin (CD79A) binding protein 1, *RPL14* = ribosomal protein L14, *FZD4* = frizzled family receptor 4, *GNS* = glucosamine (N-acetyl)-6-sulfatase, *RBCK1* = RanBP-type and C3HC4-type zinc finger containing 1.

**D.** Annotation of sAT genes associated with serum metabolic features using FunNet (Prifti *et al*. PMID: 18799481) and Gene Ontology (GO Level 6).

**E.** Known serum metabolic features associated with change in sAT gene expression.

**F.** Association of change between BCAAs and sAT genes involved in BCAA catabolism. *BCAT2* = branched chain amino-acid transaminase 2, mitochondrial; *BCKDHA* = branched chain ketoacid dehydrogenase E1, alpha polypeptide; *DBT* = dihydrolipoamide branched chain transacylase E2; *ALDH6A1* = aldehyde dehydrogenase 6 family, member A1.


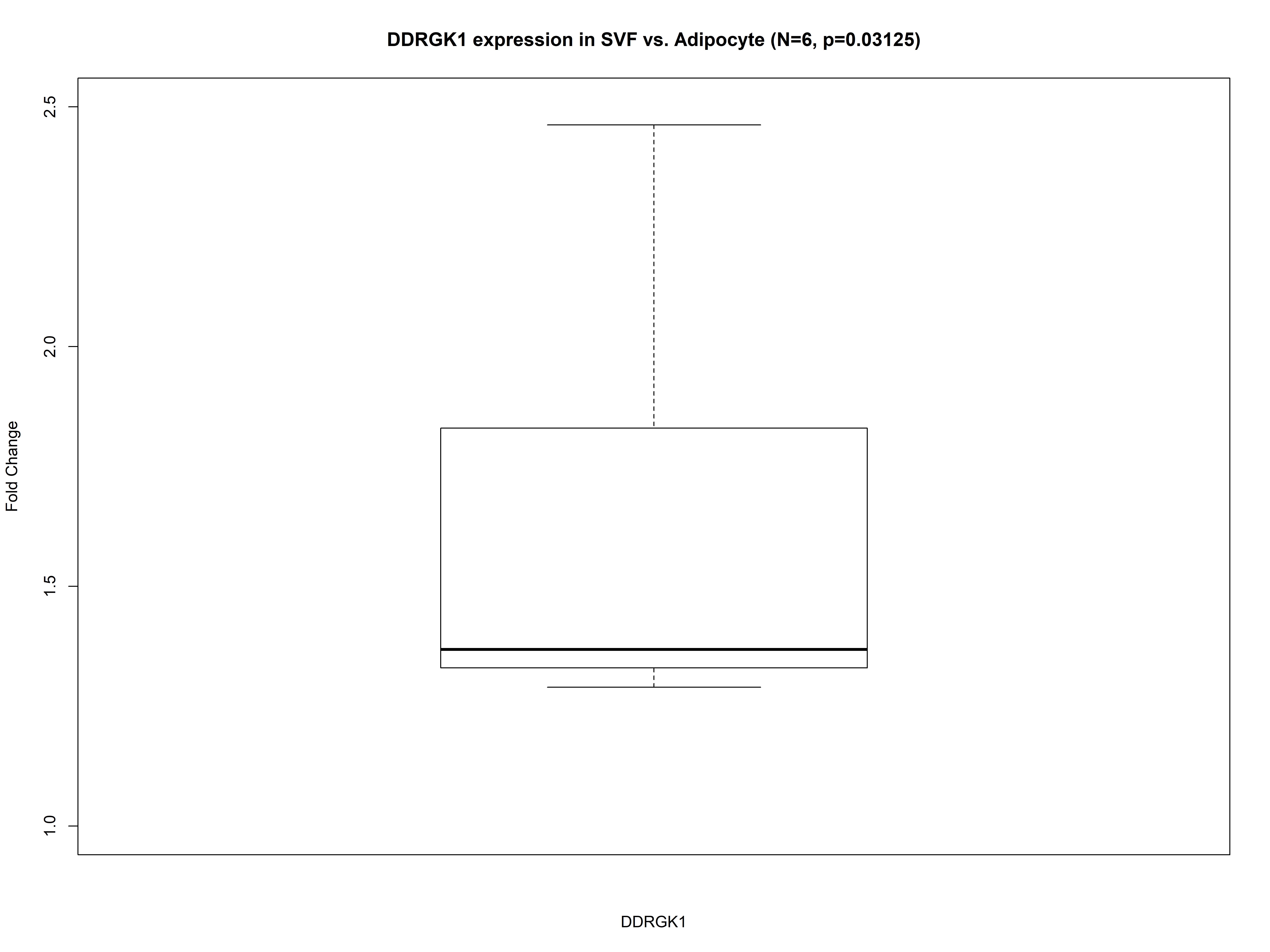


**Supplementary Figure 6. Difference in sAT *DDRGK1* expression in adipocytes versus stromal vascular fraction.**

Gene expression in subcutaneous fat (periumbilical fat) from 6 obese patients was examined. Stroma vascular fraction was isolated from adipocytes and microarrays were performed as described in Henegar *et al* (PMID: 18208606).


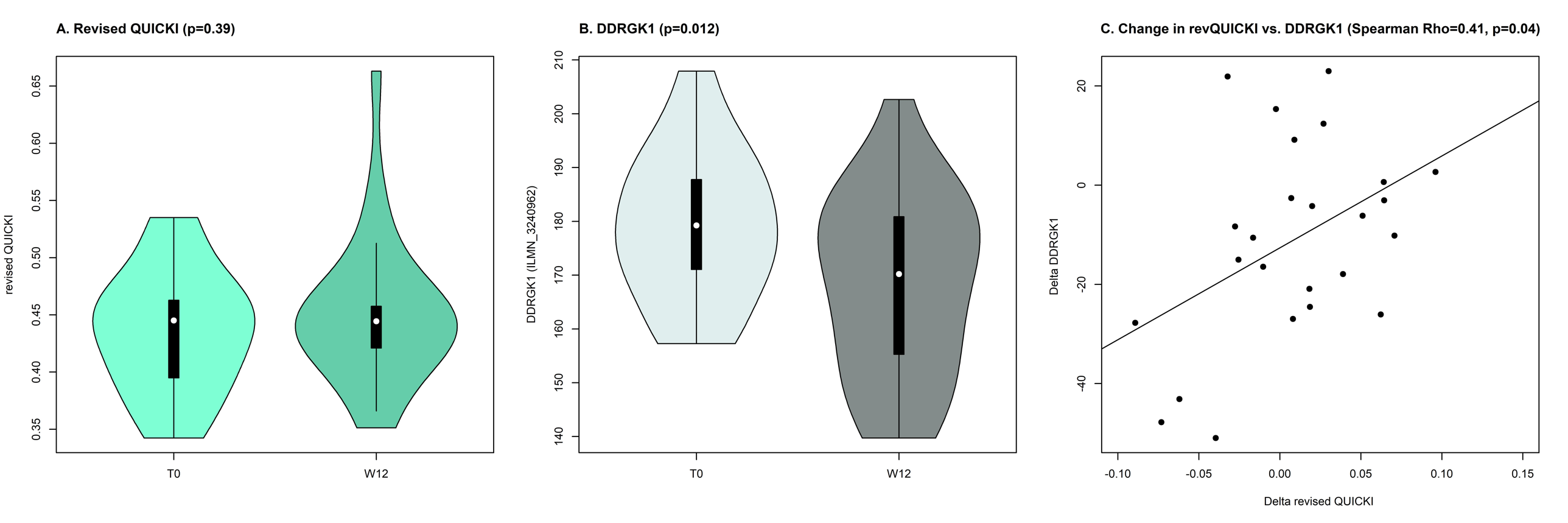


**Supplementary Figure 7. Association between sAT *DDRGK1* expression and revised QUICKI in a separate group of overweight/obese adults.**

**A, B.** Change in revised QUICKI and *DDRGK1* was assessed with Wilcoxon signed rank sum test.

**C.** Spearman correlation analysis between change in revised QUICKI and *DDRGK1*.
